# NS398 as a potential drug for autosomal‐dominant polycystic kidney disease: Analysis using bioinformatics, and zebrafish and mouse models

**DOI:** 10.1111/jcmm.16903

**Published:** 2021-09-22

**Authors:** Sixiu Chen, Linxi Huang, Shoulian Zhou, Qingzhou Zhang, Mengna Ruan, Lili Fu, Bo Yang, Dechao Xu, Changlin Mei, Zhiguo Mao

**Affiliations:** ^1^ Division of Nephrology Kidney Institute of People’s Liberation Army (PLA) Changzheng Hospital Second Military Medical University Shanghai China; ^2^ Graduate School of Clinical Medicine Second Military Medical University Shanghai China; ^3^ Internal Medicine Ⅲ (Nephrology and Endocrinology) Naval Medical Center of PLA Second Military Medical University Shanghai China

**Keywords:** autosomal‐dominant polycystic kidney disease, bioinformatics analysis, clear‐cell renal cell carcinoma, cystogenesis, NS398

## Abstract

Autosomal‐dominant polycystic kidney disease (ADPKD) is characterized by uncontrolled renal cyst formation, and few treatment options are available. There are many parallels between ADPKD and clear‐cell renal cell carcinoma (ccRCC); however, few studies have addressed the mechanisms linking them. In this study, we aimed to investigate their convergences and divergences based on bioinformatics and explore the potential of compounds commonly used in cancer research to be repurposed for ADPKD. We analysed gene expression datasets of ADPKD and ccRCC to identify the common and disease‐specific differentially expressed genes (DEGs). We then mapped them to the Connectivity Map database to identify small molecular compounds with therapeutic potential. A total of 117 significant DEGs were identified, and enrichment analyses results revealed that they are mainly enriched in arachidonic acid metabolism, p53 signalling pathway and metabolic pathways. In addition, 127 ccRCC‐specific up‐regulated genes were identified as related to the survival of patients with cancer. We focused on the compound NS398 as it targeted DEGs and found that it inhibited the proliferation of *Pkd1* ^−/−^ and 786‐0 cells. Furthermore, its administration curbed cystogenesis in *Pkd2* zebrafish and early‐onset *Pkd1*‐deficient mouse models. In conclusion, NS398 is a potential therapeutic agent for ADPKD.

## 1 INTRODUCTION

Autosomal‐dominant polycystic kidney disease (ADPKD) is the most common inherited kidney disease with a prevalence of approximately 1 in 1000 to 2500.^1^ In addition to its main clinical characteristic of uncontrolled progression of renal cysts, other manifestations include hypertension, neural aneurysm, cardiac valvular disease and metabolic disorders; thus, it is recognized as a systemic disease.^2^, ^3^, ^4^ Most patients develop symptoms at the age of 60 years, and renal function declines as the disease progresses. By the age of 70 years, approximately 70% of patients require renal replacement therapy.^5^ Although tolvaptan, the first FDA‐approved drug for ADPKD, has demonstrated efficacy in slowing the decline rate of renal function, its high price and adverse effects limit its use.^6^

ADPKD is mainly caused by mutations in *Pkd1* (85%) and *Pkd2* genes, which encode polycystin 1 (PC1) and polycystin 2 (PC2) proteins, respectively. When PC1 or PC2 is missing, the levels of intracellular calcium ions decrease, whereas those of cyclic AMP increase. This affects downstream signalling pathways, cell proliferation and the secretion of cyst fluid, ultimately leading to the formation and growth of cysts.^7^, ^8^, ^9^ The hyperproliferation of the cyst‐lining epithelium is an important hallmark of ADPKD. The main mechanisms for the excessive proliferation of cells are as follows: (1) the activation of proliferation‐related pathways, including mitogen‐activated protein kinase (MAPK)/extracellular regulated protein kinases (ERK), phosphoinositide 3‐kinase (PI3K)/Akt and mammalian target of rapamycin (mTOR) pathways; and (2) the suppression of the inhibition of cell proliferation due to loss‐of‐function mutations in PC1 and PC2.^8^, ^9^

Previous studies have found that ADPKD and tumours share many mechanisms and signalling pathways; thus, ADPKD has been called ‘neoplasia in disguise’.^10^, ^11^ Although some studies have reported a lower risk of cancer in patients with ADPKD, presumably owing to differences in detection methods, follow‐up durations and sample sizes, it is widely accepted that patients with ADPKD have a higher risk of tumours, especially kidney tumours.^12^, ^13^, ^14^, ^15^, ^16^, ^17^ Among the types of kidney tumours, clear‐cell renal cell carcinoma (ccRCC) is the most frequent histological subtype, accounting for 75% of all kidney tumour cases.^2^ Considering the similarities between ADPKD and cancer, some researchers have proposed that the therapeutics applied in cancer may be repurposed in ADPKD. Moreover, a better understanding of the critical processes that are similar between ADPKD and cancer can promote the repurposing of cancer therapeutics for ADPKD.^9^

We have previously developed the Renal Gene Expression Database (RGED), a bioinformatics database comprising comprehensive gene expression datasets of studies on renal diseases published in the NCBI Gene Expression Omnibus (GEO) database.^18^ The Connectivity Map database (CMap), which shows the relationship among diseases, genetic perturbation and drug action, was constructed by Lamb et al. in 2006.^19^ CMap contains a reference database that includes gene expression profiles derived from cultured human cells treated with multiple perturbagens. It provides a method to screen a ranked list of small molecular compounds based on the connectivity scores, which are calculated by the Kolmogorov–Smirnov statistic, by comparing disease gene expression patterns with the reference database. A significantly negative correlation between the disease‐related gene expression pattern and the gene profile after compound treatment suggests that the compound has therapeutic potential for the disease. Therefore, using the CMap database, we can discover new indications for existing drugs.^19^, ^20^, ^21^, ^22^

To clarify the similarities and dissimilarities between ADPKD and ccRCC and screen and identify candidates for ADPKD therapy, we designed an integrated bioinformatics approach based on the aforementioned rationale and conducted corresponding experiments for validation. Thus, our results will provide insights into the relationship between ADPKD and ccRCC, as well as a new therapeutic strategy for ADPKD.

## 2 MATERIALS AND METHODS

### 2.1 Microarray datasets

We collected the gene expression profiles of renal cystic tissues of patients with ADPKD and tumour tissues of patients with ccRCC from RGED (http://rged.wall‐eva.net) and the high‐throughput gene expression datasets from GEO (http://www.ncbi.nlm.nih.gov/geo/). The two datasets retrieved were GSE7869 and GSE53757, both constructed based on the GPL570 platform (Affymetrix Human Genome U133 Plus 2.0 Array). GSE7869 included cystic tissues of different sizes from 5 *Pkd1* polycystic kidneys and non‐cancerous renal cortical tissues from 3 nephrectomized kidneys, whereas GSE53757 included 72 ccRCC tumour tissues of all disease stages and matched kidneys.

### 2.2 Data processing and differentially expressed gene (DEG) analysis

R packages simpleaffy, affyPLM, affy, gcrma and limma (https://www.r‐project.org, v3.5.1) were used for background correction, standardization and expression value calculation of the original datasets GSE7869 and GSE53737. Fold‐change and adjusted *p*‐values were used to screen DEGs. The annotate package was utilized to annotate DEGs. Volcano maps of DEGs were constructed using ggplot2. Subsequently, we identified intersecting DEGs between the datasets using the Venn package in R.

### 2.3 Function enrichment, protein–protein interaction (PPI) network and survival analysis

Function enrichment and Kyoto Encyclopedia of Genes and Genomes (KEGG) pathway enrichment analyses were performed using the Database for Annotation, Visualization and Integrated Discovery 6.8 (https://david.ncifcrf.gov) to investigate the function of the DEGs. Gene ontology (GO) annotation includes three categories: biological process (BP), cellular component (CC), and molecular function (MF). Significant GO terms and KEGG pathways were defined as *p* < 0.05. The interaction between proteins was identified using the String database (http://string‐db.org, v10.0). Kaplan‐Meier plots and log‐rank test results were retrieved using the cgdsr, survival and survminer package in R. The high‐expression group was defined as those with expression value > 75% quantile, whereas the low‐expression group was defined as those with expression value < 25% quantile. *p* < 0.01 was set as the cut‐off value.

### 2.4 Connectivity Map analysis

The CMap (build 02, https://portals.broadinstitute.org/CAMP/) provides gene expression profiles from cultured human cells treated with bioactive small molecules and can be used to discover functional connections among diseases, genetic perturbation and drug action.^22^ Therefore, we queried the connectivity scores between the profile of interest and the instances in the database based on the Kolmogorov‐Smirnov statistic. The connectivity score is a value between −1 and +1, similar to correlation coefficients, with a negative score representing a counteracting effect of the compound on the expression pattern of the input profile, which in this case was the common DEGs. The cut‐off value was defined as a connectivity score < −0.7 and *P* value < 0.05.

### 2.5 Cell culture and drug treatment

ADPKD cyst‐lining epithelial mouse cells (herein, *Pkd1* ^−/−^ cells) were a kind gift from Yiqiang Cai (Yale University School of Medicine) and cultured as described by Shibazaki et al.^23^ The human renal clear‐cell adenocarcinoma cell line 786‐0 was purchased from Shanghai Institute of Cell Biology and grown in Dulbecco's modified Eagle's medium/Nutrient Mixture F‐12 (Gibco/Life Technologies) supplemented with 10% foetal bovine serum (Gibco/Life Technologies) at 37°C. NS398 was purchased from Selleck and dissolved in dimethylsulfoxide (DMSO; Amresco) to prepare a stock solution. Both cells were treated with NS398 at 50 or 100 μM for 24 or 48 h, respectively. The control group was administered the same volume of DMSO.

### 2.6 Methylthiazolyldiphenyl‐tetrazolium bromide (MTT) assays

*Pkd1* ^−/−^ and 786‐0 cells were seeded in culture plates and incubated for 12 h at 33 and 37°C, respectively. NS398 was then administered for 1, 2 or 3 days, respectively. DMSO was used as the vehicle for the compound. After treatment with MTT (Invitrogen‐Life Technology) for 4 h, DMSO was added to dissolve the crystallized products. Absorbance at 490 nm was measured. The measurement was repeated in six duplicate wells.

### 2.7 Cell cycle analyses

Cell cycle staining was performed using a propidium iodide (PI) cell cycle kit according to the manufacturer's instructions (Multi Sciences Biotech). Briefly, cells were harvested, washed twice with phosphate‐buffered saline and stained with 50 µg/ml PI. Cells were then counted in a flow cytofluorometer based on red emissions at 630 nm, and data were analysed using a Beckman Coulter CyAn ADP analyzer.

### 2.8 EdU staining

EdU assays were conducted following the manufacturer's instructions (Beyotime). After adding 2× EdU working solution to each well, 24‐well plates were placed in the incubator (33°C for *Pkd1* ^−/−^ or 37°C for 786–0 cells) for 4 h. After fixing with 4% formaldehyde, the click reaction cocktail (0.2 ml) was added to each well, and the DNA was stained with Hoechst. The samples were imaged using Olympus BX‐51 fluorescence microscopy.

### 2.9 Western blot analyses

A column tissue and cell protein extraction kit (Minute) was used to extract the proteins of cultured and processed cells and mouse kidney tissues. Protein was quantified using a BCA kit (Thermo Fisher Scientific) following the manufacturer's instructions. The protein concentration of each sample was balanced by double‐distilled H_2_O (ddH_2_O) and 5× protein loading buffer (Yamei) was added to the protein sample. The solution was boiled in a 95°C dry heater for 15 min. An equal amount (20 μl) of protein was loaded onto an SDS‐PAGE gel and electrophoresed, and separated proteins were transferred onto a polyvinylidene fluoride membrane. After incubation with the appropriate primary and secondary antibodies, development was performed using an enhanced chemiluminescence reagent (Thermo Fisher Scientific). Densitometric analysis for the determination of relative protein expression was performed using Image lab system (Bio‐Rad Laboratory), with GAPDH as a loading control.

### 2.10 Animal studies

Wild‐type zebrafish (Nüsslein‐Volhard Laboratory) were maintained, mated, and staged as described previously.^24^ *Pkd2* morpholino (*Pkd2* MO): 5ʹ‐AGGACGAACGCGACTGGAGCTCATC‐3ʹ was constructed using Gene Tools (Philomath) and injected at the 1‐cell stage.^25^ A total of 100 *Pkd2* morphants (knockdown animals generated by morpholino antisense oligo) were divided into two groups 24 h post‐fertilization. One group was treated with NS398 (10 μM), whereas the other was treated with DMSO as the vehicle‐treated control group. The embryos were anaesthetized and immobilized in methylcellulose 72 h post‐fertilization to visualize the pronephric cyst formation and measure the rate of cyst formation in all embryos. The experiment was independently conducted three times.

The early‐onset PKD mouse model was generated as described previously.^26^ Briefly, tamoxifen (10 mg/kg) was intraperitoneally injected into C57/BL6 *Pkd1*fl/fl: tamoxifen‐Cre mice at postnatal day 10 (P10) to induce *Pkd1* deficiency. NS398 was administered every other day via intraperitoneal injection from P13 at 20 μg/g body weight. Mice were killed by isoflurane using a rodent gas anaesthesia machine (Yuyan Machine) at P30; blood was collected by retro‐orbital puncture and kidneys were harvested. The serum blood urea nitrogen (BUN) concentration was measured with a urea assay kit (BioAssay Systems). The kidneys were weighted and collected for morphological analysis and Western blot analysis.

The study protocol was approved by the Second Military Medical University's Animal Care and Use Committee (approval number SMMU‐ACUC‐20150430).

### 2.11 Morphological analysis

Kidney sections were fixed in 4% paraformaldehyde buffered solution and embedded in paraffin. Then kidneys were sliced into 2‐μm‐thick sections and stained with haematoxylin and eosin (HE). Aperio XT and Leica Aperio (Leica Biosystems) were applied to scan all specimens. The size and shape of kidney cysts were quantitatively measured from the sagittal section of the kidney. The cyst index (CI) was determined as previously described by Yang et al.^26^ Briefly, the whole kidney image was divided into 20‒30 small regions and the cystic area in each split image and the entire kidney area were measured using Image Pro Plus (v6.0). The CI was determined by dividing the sum of the cystic area by the whole kidney area.

### 2.12 Statistical analyses

Data are presented as the mean ± standard error of at least triplicates or as a representative of three separate experiments. Significance was determined using a two‐sided Student’s *t* test. *p* < 0.05 was considered statistically significant.

## 3 RESULTS

### 3.1 Identification of DEGs from GEO datasets

**FIGURE 1:**
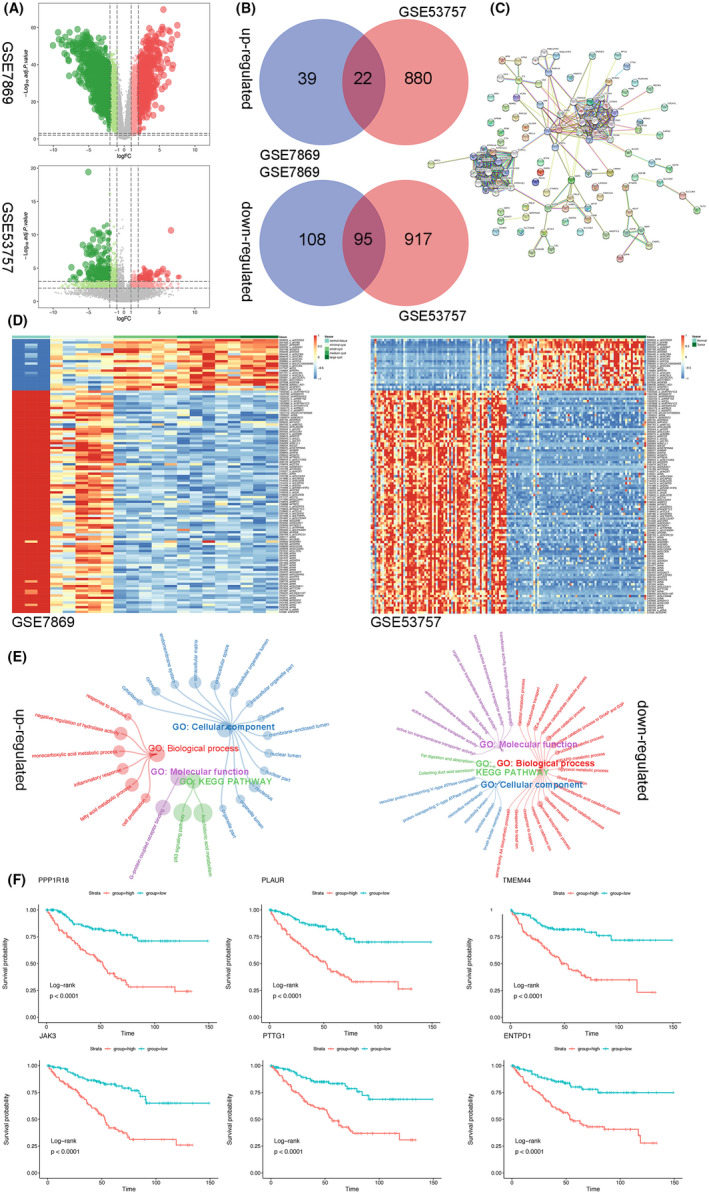
Differentially expressed genes (DEGs) identified in the GSE7869 and GSE53757 datasets. (A) Volcano maps of DEGs in GSE7869 and GSE53757. (B) Venn diagrams displaying the commonly up‐regulated and down‐regulated DEGs. (C) Protein–protein interaction network of common DEGs. (D) Heatmap of up‐regulated and down‐regulated DEGs. The coloured bars on the top (shades of green) indicate the sample types. (E) GO and KEGG pathway enrichment analysis of common DEGs. (F) Kaplan‐Meier survival analysis of the top 6 unique DEGs in ccRCC ranked by hazard ratio based on the TCGA Kidney (Renal Cell) Cancer data (the red line represents high‐expression group, whereas the blue line represents low‐expression group)

Detailed information about the GEO datasets is summarized in Table 1. The volcano plot of the screened DEGs is displayed in Figure 1A. We identified 61 up‐regulated and 203 down‐regulated DEGs in GSE7869 and 902 up‐regulated and 1012 down‐regulated DEGs in GSE53757 by setting the cut‐off value as |log_2_FC| ≥ 2 and *p* < 0.001. From the Venn diagrams, 22 up‐regulated and 95 down‐regulated DEGs were found to be common to both datasets (Figure 1B). From the PPI network graph, two modules were related to cell cycle and energy metabolism (Figure 1C). Heatmaps showed the expression profiles of the common DEGs (Figure 1D).

**TABLE 1:** Tissue types/samples included in GSE7869 and GSE53757 datasets

GEO	Disease	Platform	Sample size	Experimental (*n*)	Control (*n*)
GSE7869	ADPKD	GPL570	21	Small cysts (5)	Normal renal cortical tissue (3)
				Medium cysts (5)	
				Large cysts (3)	
				Minimally cystic Tissue (5)	
GSE53757	ccRCC	GPL570	144	Stage 1 (24)	Matched normal kidney (24)
				Stage 2 (19)	Matched normal kidney (19)
				Stage 3 (14)	Matched normal kidney (14)
				Stage 4 (15)	Matched normal kidney (15)

Note

small cysts: less than 1 ml; medium cysts: between 10 and 25 ml; large cysts: greater than 50 ml; ccRCC stages were defined based on pathology reports.

Common up‐regulated DEGs were enriched in stimulus‐response, fatty acid metabolism, negative regulation of hydrolase activity, cell proliferation, monocarboxylic acid metabolism and inflammatory response for BP; extracellular matrix and nucleolus for CC; G protein‐coupled receptor binding for MF. KEGG pathway analysis results revealed that the common up‐regulated DEGs were significantly enriched in arachidonic acid metabolism and the p53 signalling pathway. The overlapping down‐regulated DEGs were mainly enriched in catabolic process and transport for BP; ATPase complex for CC; and transporter activities for MF. In addition, the overlapping down‐regulated DEGs mainly participated in metabolic pathways and carbon metabolism (Figure 1E). These results indicated that these signalling pathways, shared by ADPKD and ccRCC, cause the similarities between the diseases.

Because the main difference between ADPKD and ccRCC is the malignant and metastatic nature of ccRCC, which determines the prognosis of patients, we have been suggested that ccRCC‐specific DEGs are closely related to the prognosis of patients with ccRCC. Of the 880 genes, 127 were identified to be significantly related to the survival of patients with kidney cancer (log‐rank, *p* < 0.01). The top 6 genes were *PPP1R18*, *PLAUR*, *TMEM44*, *JAK3*, *PTTG and ENTPD1* ranked by the hazard ratio, and their Kaplan‐Meier survival curves are displayed in Figure 1F.

### 3.2 Connectivity Map identified NS398 as a potential therapeutic compound

Using the CMap database, three compounds were identified as potential compounds that reverse the transcriptional changes shared by ADPKD and ccRCC, as they had negative mean connectivity scores (Table 2). Among them, the drug NS398, which has been studied in laboratory experiments, has an inhibitory effect on tumour progression. Thus, it was selected as a novel drug for further studies to confirm its value in ADPKD treatment.

**TABLE 2:** Small molecules identified in Connectivity Map

CMap name	Mean connectivity scores	*N*	*P* value	Non‐null (%)
1,4‐chrysenequinone	−0.638	2	0.00324	100
arachidonic acid	−0.618	3	0.00214	100
NS398	−0.538	3	0.00405	100

Note

N represents the sample size of instances related to the compounds in the database. The connectivity scores and *P* value were calculated based on the Kolmogorov‐Smirnov statistic.

### 3.3 NS398 inhibits cell proliferation *in vitro*

**FIGURE 2:**
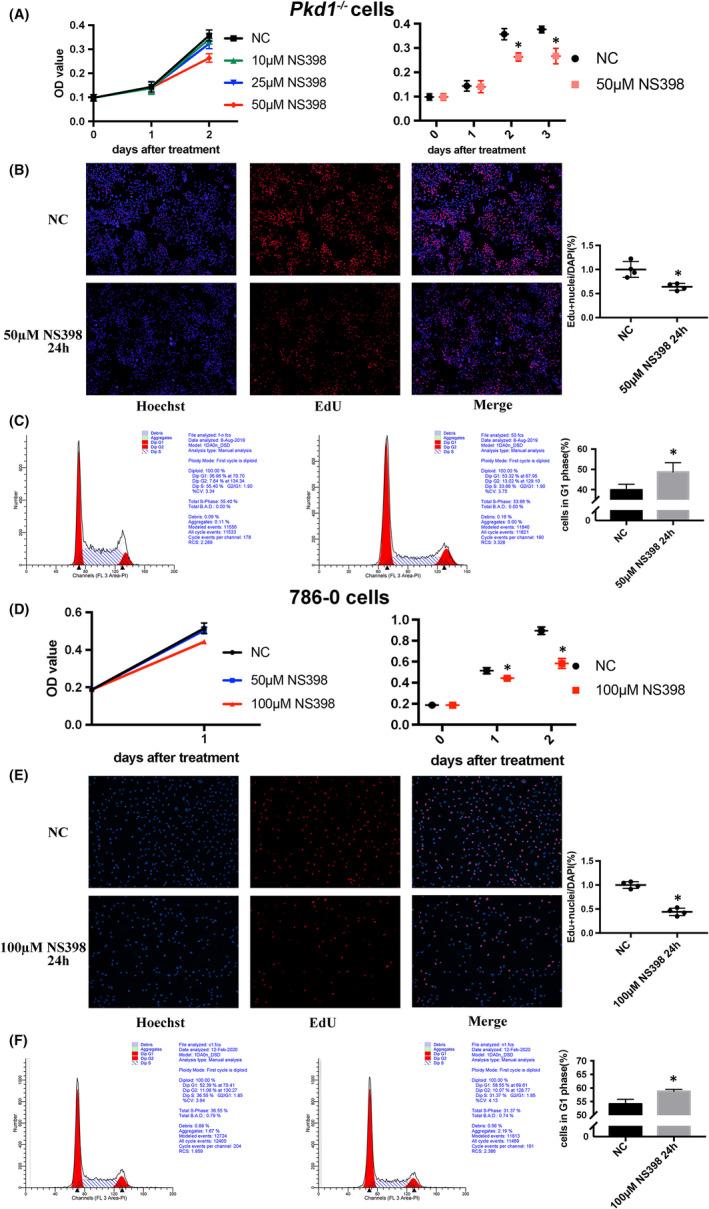
NS398 inhibits *Pkd1* ^−/−^ and 786‐0 cell proliferation *in vitro*. (A) MTT assay of *Pkd1* ^−/−^ cells after administration of NS398 at different doses. (B) EdU proliferation assay analysis of the growth of *Pkd1* ^−/−^ cells after treatment with 50 μM NS398 for 24 h. The cells in blue (stained with Hoechst) represent all living cells, whereas the red fluorescent cells are in the S phase of mitosis. (C) Effects of 50 μM NS398 on cell cycle distribution of *Pkd1* ^−/−^ cells. (D) MTT assay of 786‐0 cells after administration of NS398 at different doses. (E) and (F) EdU proliferation assay and cell cycle analyses of the effects of 100 μM NS398 on the growth of 786‐0 cells after treatment for 24 h (* *p* < 0.05, *t* test)

We observed an inhibitory effect on cell proliferation in both *Pkd1* ^−/−^ *and* 786‐0 cells after NS398 treatment in a dose‐ and time‐dependent manner. The effective dose for *Pkd1* ^−/−^ cells was 50 μM, and a decrease in cell viability was observed after treatment for 2 or 3 days (Figure 2A, *p* < 0.05). Consistent with the results of the MTT assays, the percentage of EdU‐positive cells was lower in *Pkd1* ^−/−^ cells treated with 50 μM NS398 than that in cells treated with DMSO (Figure 2B). Treatment with 50 μM NS398 for 24 h induced cell cycle arrest at the G1 phase in *Pkd1* ^−/−^ cells and significantly increased the number of cells in the G1 phase (from 39.86 ± 1.62% to 48.74 ± 2.61%, *p* < 0.05; Table 3; Figure 2C). NS398 treatment did not induce apoptosis in *Pkd1* ^−/−^ cells as Annexin V + PI staining did not show a marked difference in the number of apoptotic cells after treatment with 50 μM NS398 for 24 h (Figure S1).

**TABLE 3:** Proportion of *Pkd1* ^−/−^ and 786‐0 cells in each cell cycle phase after NS398 administration

Cell type	Treatment	G1 phase (%)	S phase (%)	G2 phase (%)
*Pkd1^−/−^*	DMSO	39.86 ± 1.62	52.95 ± 3.62	8.19 ± 1.81
	NS398 (50 μM)	48.74 ± 2.61*	41.31 ± 4.44	9.95 ± 1.84
786–0	DMSO	54.13 ± 0.97	36.63 ± 0.21	9.24 ± 1.12
	NS398 (100 μM)	58.79 ± 0.41*	30.87 ± 0.48	10.41 ± 0.17

Note

These data are mean ± SD of three individual experiments.

* Means *p *< 0.05 compared to the vehicle DMSO (*t* test).

Likewise, NS398 exhibited an inhibitory effect on the proliferation of 786‐0 cells. Cell viability significantly decreased at 100 μM as confirmed by MTT assays (Figure 2D, *p* < 0.05). The EdU proliferation assay showed a similar result—the number of EdU‐positive 786‐0 cells was remarkably reduced after treatment with 100 μM NS398 (Figure 2E). Cell cycle analysis indicated G1 phase arrest in 786‐0 cells after treatment with 100 μM NS398 (Table 3; Figure 2F).

### 3.4 NS398 down‐regulates cell cycle proteins and proliferation‐related signalling pathways

**FIGURE 3:**
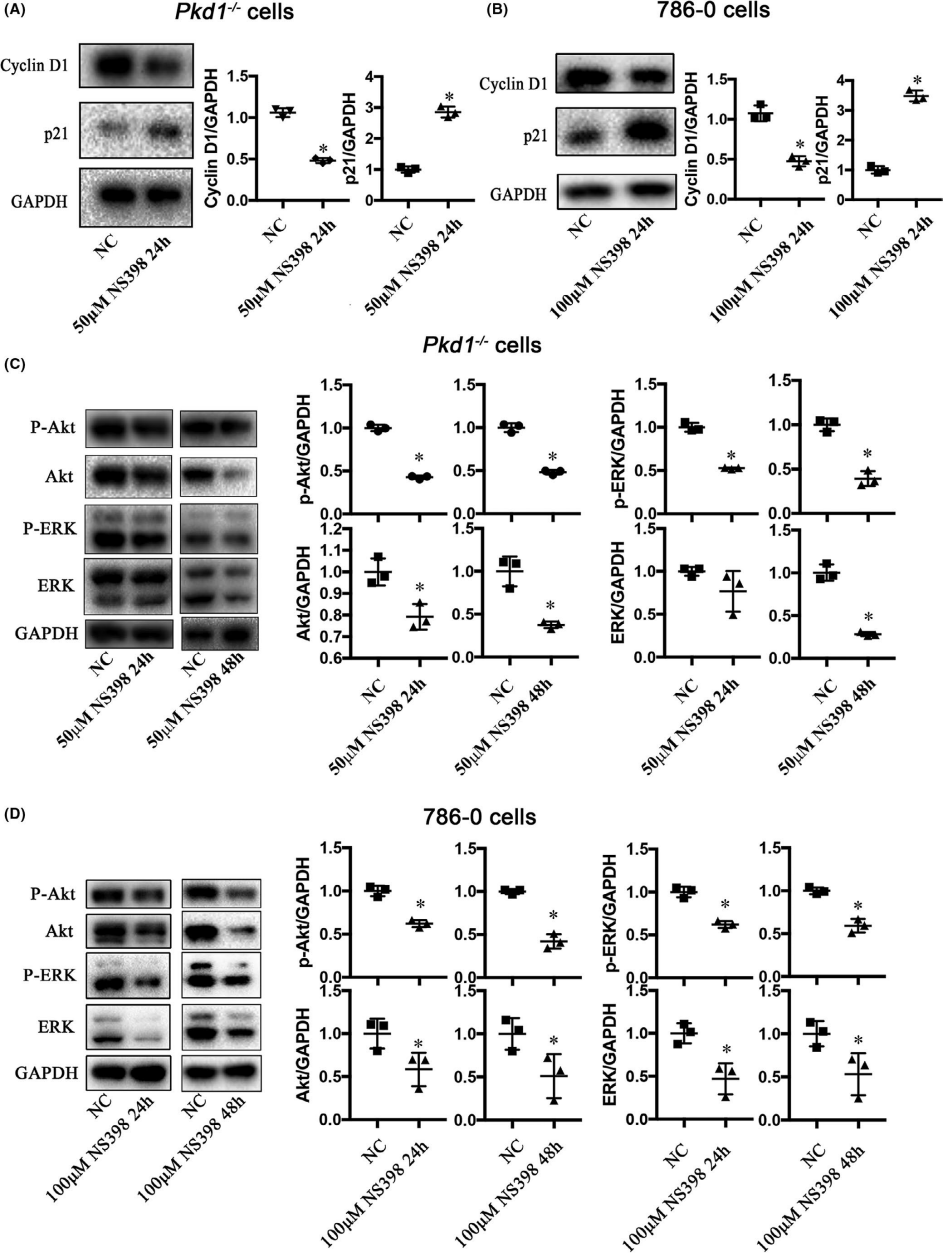
NS398 down‐regulates the abundance of cell cycle and proliferation‐related signalling proteins. (A) and (B) Western blot showing the effects of NS398 on the levels of cyclin D1 and p21 in *Pkd1* ^−/−^ (50 μM) and 786‐0 cells (100 μM), respectively. (C) and (D)Western blot showing the effects of NS398 on the levels of phospho‐ and total Akt and ERK in *Pkd1* ^−/−^ (50 μM) and 786‐0 (100 μM) cells for 24 and 48 h, respectively. GAPDH was used as the loading control (*p < 0.05, *t* test)

The abundance of cyclin D1 and p21, as representative proteins involved in the cell cycle, was detected. Treatment with NS398 reduced the levels of cyclin D1 but increased those of p21 in both *Pkd1* ^−/−^ and 786‐0 cells at concentrations of 50 and 100 μM, respectively (Figure 3A,B).

PI3K/Akt and ERK pathways regulate cell proliferation and cell cycle. Western blot analysis showed that NS398 treatment (at 50 or 100 μM) reduced the levels of phospho‐ and total Akt and ERK in *Pkd1* ^−/−^ and 786‐0 cells (Figure 3C,D). NS398 inhibited the expression of downstream cell cycle‐related proteins by inactivating the PI3K/Akt and ERK signalling pathways, thereby arresting the cell cycle in the G1 phase and exerting an anti‐cell proliferation effect.

### 3.5 NS398 attenuates the progression of kidney cyst formation *in vivo*

**FIGURE 4:**
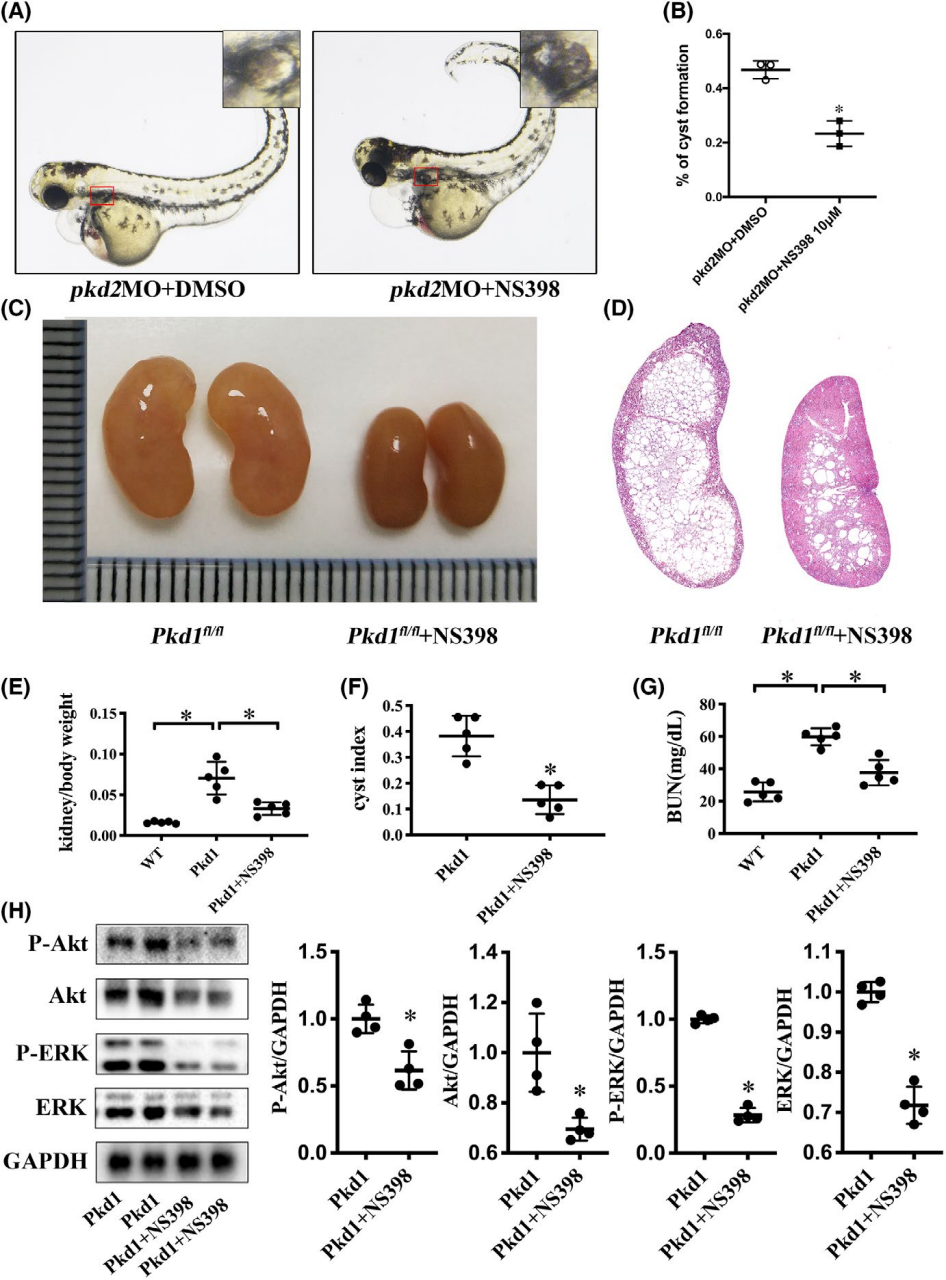
NS398 reduces cyst formation *in vivo*. (A) Representative images of *Pkd2* morphants treated with vehicle (DMSO) or NS398. (B) Quantification showing the average percentage of embryos with kidney cysts in the two groups from three independent experiments. Fifty embryos per group were examined in each experiment. (C) and (D) Representative immunohistochemical images of kidneys and scans of whole kidneys isolated from *Pkd1*‐knockout mice treated with the vehicle (DMSO) or 20 μg/g NS398, stained with HE. (E), (F), and (G) Quantitative analysis of kidney/body weight ratio, CI, and BUN levels of wild‐type, diseased and NS398‐treated mice (*n* = 5 per group; *p* < 0.05.) (H) Western blot showing the effects of NS398 on the levels of phospho‐ and total Akt and ERK in diseased and NS398‐treated mice (*p < 0.05, *t* test)

Representative images of cyst formation in the *Pkd2* morphants administered DMSO or NS398 are shown in Figure 4A. Cyst formation was visualized under a microscope (5×) in both groups; however, the percentage of cyst formation in the *Pkd2* morphants significantly decreased after treatment with 10 μM NS398 (46.9% in DMSO‐treated morphants *vs* 23.4% in NS398‐treated morphants, *p* < 0.05; Figure 4B).

Similarly, we observed the attenuation of cystogenesis in early‐onset *Pkd1* conditional knockout mice. NS398 treatment remarkably attenuated the increase in kidney size in early‐onset *Pkd1* conditional knockout mice (Figure 4C,D). The kidney weight‐to‐body weight ratio (Figure 4E) and the CI (Figure 4F) in NS398‐treated mice were both markedly reduced. The decreased rate of cyst growth was accompanied by improved renal function, as evidenced by BUN levels (Figure 4G). Western blot analysis indicated that the levels of phospho‐ and total Akt and ERK significantly decreased in NS398‐treated *Pkd1* conditional knockout mice (Figure 4H).

Therefore, NS398 inhibited cyst growth in both *Pkd2* zebrafish and early‐onset *Pkd1*‐deficient mouse models and improved the renal function in early‐onset *Pkd1*‐deficient mouse model.

## 4 DISCUSSION

In this study, we searched the RGED and NCBI‐GEO databases to retrieve datasets pertaining to ADPKD and ccRCC and conducted a comprehensive bioinformatics analysis to unravel their convergences and divergences. Although many studies have investigated the similarities and dissimilarities between ADPKD and ccRCC using laboratory studies, to the best of our knowledge, this is the first report to unravel their links using high‐throughput research. Thus, our findings provide a better understanding of their critical mechanisms.

Our results showed that upregulated common DEGs are mainly enriched in arachidonic acid metabolism and the p53 signalling pathway, whereas downregulated DEGs are enriched in metabolic pathways and carbon metabolism. The PPI results of common genes indicated that two modules pertain to the cell cycle and energy metabolism. Several cancer studies have demonstrated that the arachidonic acid pathway promotes cell proliferation, and that its suppression is a potential target for cancer prevention.^27^, ^28^, ^29^ In addition, Klawitter et al.^30^ have reported that the level of 20‐HETE, a metabolite of arachidonic acid, is significantly increased in the serum samples of patients with ADPKD and correlates with estimated glomerular filtration rate and total kidney volume. The p53 signalling pathway plays a determinant role in oncogenesis and has recently been revealed as a master regulator in ADPKD progression via interaction with Wnt/beta‐catenin, c‐Jun N‐terminal kinase and MAPK signalling pathways.^31^, ^32^, ^33^, ^34^ In our study, treatment with NS398, an inhibitor of arachidonic acid metabolism, arrested the cell cycle in both *Pkd1* ^−/−^ and 786‐0 cells, which is consistent with the bioinformatics results.

The most significant difference between ADPKD and cancer is the invasive and metastatic nature of cells observed in the latter. Most deaths in patients with cancer are related to uncontrolled invasion and metastasis, which is a complex process.^35^ Therefore, we also analysed the specific upregulated DEGs in ccRCC and found that the high expression of *PPP1R18*, *PLAUR*, *TMEM44*, *JAK3*, *PTTG1* and *ENTPD1* was related to the poor prognosis of patients. *PLAUR*, *JAK3*, *PTTG1* and *ENTPD1* have been reported to be associated with ccRCC, whereas to the best of our knowledge, no study has reported the association of *PPP1R18* and *TMEM44* with ccRCC.^36^, ^37^, ^38^, ^39^, ^40^, ^41^, ^42^, ^43^, ^44^ Thus, future studies should focus on elucidating the roles of *PPP1R18* and *TMEM44* in ccRCC.

NS398 is a highly selective COX‐2 inhibitor, and previous studies have demonstrated that NS398 is less toxic to the gastric mucosa and renal papillary tissues than other COX‐2 inhibitors.^45^, ^46^, ^47^ Xu et al.^48^ reported that celecoxib, another COX‐2 inhibitor, inhibits the proliferation of cyst‐lining epithelial cells of patients with ADPKD and delays the progression of cyst formation in *Pkd1*‐knockout mice by downregulating the VEGF/Raf/MAPK/ERK signalling pathway. However, despite the evidence regarding the association between celecoxib and renal function impairment, its efficacy in ADPKD has not been validated in the clinical setting. Here, NS398 was identified based on high‐throughput bioinformatics analysis and not merely its inhibitory effect on COX‐2. Using a systematic literature study, we found that NS398 has an inhibitory effect on various tumours via COX‐2‐ and/or non‐COX‐2‐dependent signalling pathways. Lee et al.^49^ examined the effect of COX‐2 inhibitors on ageing using an intrinsic skin ageing model of hairless mice. They reported that although NS398 inhibits skin ageing and reduces the expression of p53 and p16, celecoxib and aspirin accelerate skin ageing and enhance p53 and p16 expression. Su et al.^50^ utilized N‐methyl NS398, which lacks COX‐2 inhibitory activity but is similar in structure to NS398, to suggest that NS398 decreases aromatase expression in breast cancer cells via mechanisms independent of COX‐2. Furthermore, Zhang et al.^51^ have proposed that in addition to the COX‐2‐dependent pathway, NS398 inhibits cell proliferation and induces apoptosis in colon cancer cells via the Notch1 signalling pathway. Owing to its low toxicity to kidney cells, COX‐2 independent effects and application prospects in tumours, NS398 is a promising drug for ADPKD therapy. To the best of our knowledge, this is the first study to verify the *in vitro* and *in vivo* effects of NS398 in ADPKD. Our study had some limitations. The duration of NS398 treatment was not sufficient to observe its potential long‐term effects because of the limited lifespan of the animals we used. Therefore, more studies should be conducted to investigate the effects, metabolism, efficacy and adverse effects of NS398 *in vivo*. Whether NS398 affects the functions of the liver, kidneys and other organs should also be clarified. Thus, the application of NS398 in other PKD animal models such as late‐onset *Pkd1* conditional knockout mice, *Pkd1^RC^* ^/^ *^RC^* mice and PCK rats should be considered in future studies. The results of this study suggest that NS398 exerts its inhibitory effect on cystogenesis via down‐regulation of ERK and Akt signalling pathways; however, a detailed investigation is needed to elucidate the molecular mechanisms underlying the physiological effects of this compound *in vivo*. Overall, the safety and mechanisms of action of NS398 need further exploration in the future.

In summary, we applied an integrated bioinformatics approach based on the high‐throughput data of ADPKD and ccRCC and verified these results *in vitro* and *in vivo*. We demonstrated that the small molecule compound NS398 regulates the expression of cyclin D1 and p21 via ERK and PI3K/Akt signalling pathways, thereby suppressing the proliferation of polycystic kidney cyst‐lining epithelial (*Pkd1* ^−/−^) and renal clear‐cell carcinoma (786‐0) cells. Furthermore, NS398 significantly inhibited cyst formation in zebrafish and mouse animal models of PKD. Thus, our results indicate the value of NS398 in the treatment of ADPKD and/or tumours. Basic and clinical studies are warranted to validate our results.

## CONFLICT OF INTEREST

The authors confirm that there are no conflicts of interest.

## AUTHOR CONTRIBUTION

**Sixiu Chen:** Data curation (equal); Formal analysis (equal); Investigation (equal); Methodology (equal); Software (equal); Validation (equal); Visualization (equal); Writing‐original draft (lead). **Linxi Huang:** Data curation (equal); Formal analysis (equal); Investigation (equal); Methodology (equal); Software (equal); Validation (equal); Visualization (lead); Writing‐original draft (equal). **Shoulian Zhou:** Data curation (equal); Formal analysis (equal); Investigation (equal); Methodology (equal); Validation (lead); Writing‐original draft (equal). **Qingzhou Zhang:** Data curation (equal); Formal analysis (equal); Software (lead). **Mengna Ruan:** Formal analysis (equal); Investigation (equal); Methodology (equal); Validation (equal). **Lili Fu:** Data curation (equal); Formal analysis (equal); Visualization (equal). **Bo Yang:** Data curation (equal); Formal analysis (equal); Methodology (equal). **Dechao Xu:** Data curation (equal); Formal analysis (equal); Methodology (equal); Resources (supporting); Writing‐original draft (supporting). **Changlin Mei:** Conceptualization (equal); Formal analysis (supporting); Funding acquisition (equal); Project administration (equal); Resources (equal); Supervision (equal); Writing‐review & editing (equal). **Zhiguo Mao:** Conceptualization (equal); Formal analysis (supporting); Funding acquisition (equal); Project administration (equal); Resources (equal); Supervision (equal); Writing‐review & editing (equal).

## Supporting information

Fig S1

Supinfo S1

## Data Availability

The data that support the findings of this study are publicly available at https://doi.org/10.6084/m9.figshare.13604225.v1, reference number 13604225.v1.
