# Supplementary figures and images for: NS398 as a potential drug for autosomal‐dominant polycystic kidney disease: Analysis using bioinformatics, and zebrafish and mouse models
Source: J Cell Mol Med. 2021 Sep 22;25(20):9597–608. doi: 10.1111/jcmm.16903 (PMC8505825; doi:10.1111/jcmm.16903)

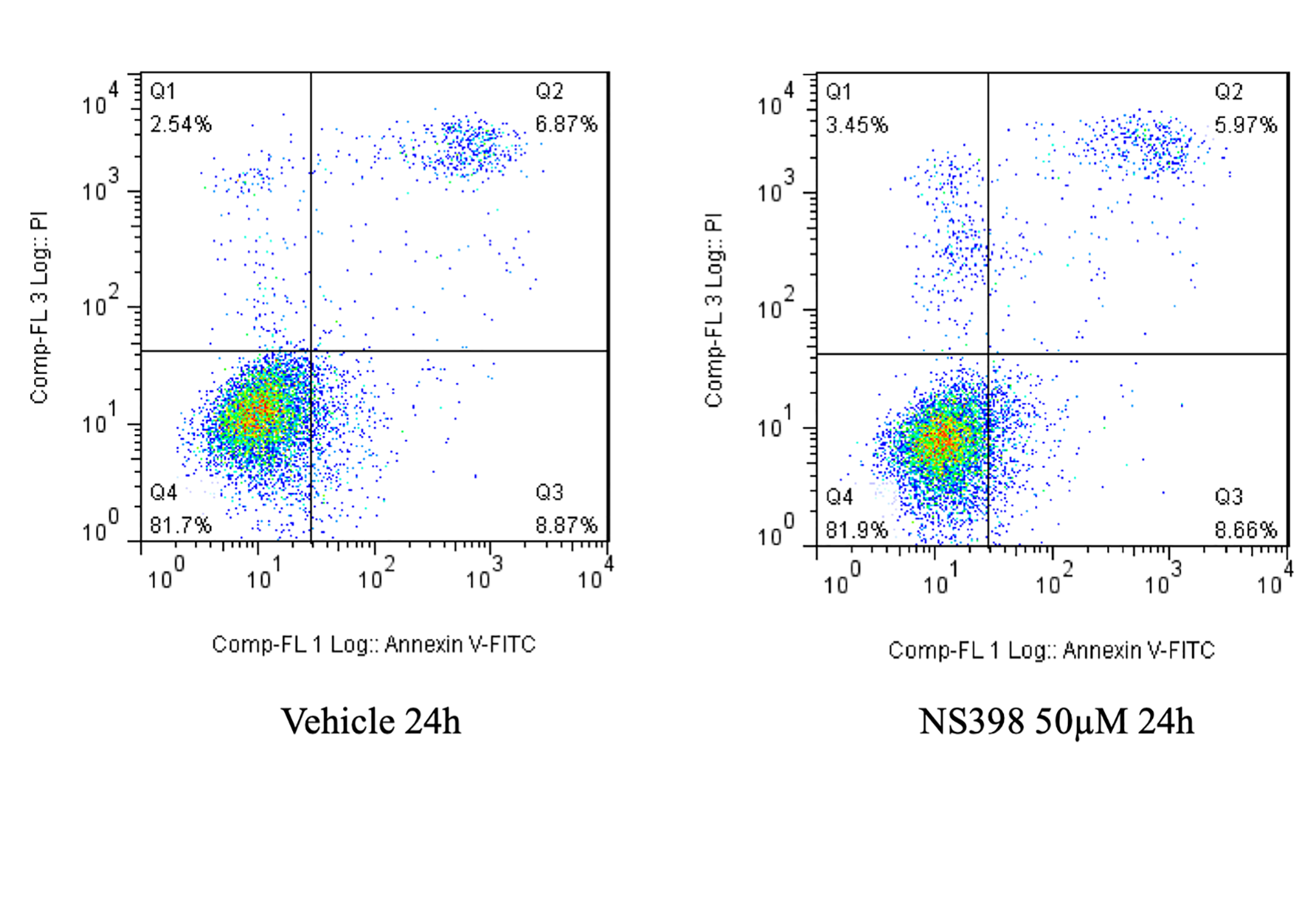

Supplement: Supplementary file 1 — Fig S1 [file JCMM-25-9597-s002.tif]
