# Supplementary material for: NS398 as a potential drug for autosomal‐dominant polycystic kidney disease: Analysis using bioinformatics, and zebrafish and mouse models
Source: J Cell Mol Med. 2021 Sep 22;25(20):9597–608. doi: 10.1111/jcmm.16903 (PMC8505825; doi:10.1111/jcmm.16903)
Supplement: Supplementary file 2 — Supinfo S1 [file JCMM-25-9597-s001.docx]

**Supplementary Materials and Methods:**

**Cell apoptosis assay**

Cells were seeded in 6 cm culture plates and treated with vehicle and 50 µM NS398 for 24 h. Then cells were harvested and performed cell apoptosis assay following the manufacturer’s instructions (Annexin V-FITC/PI apoptosis kit, AP101, Multi Sciences Biotech, China). In brief, cells were re-suspended with binding buffer and stained with propidium iodide (PI) and Annexin V-FITC for 5 minutes. After that, flow cytometry was carried out to detect apoptotic cells.

**Supplementary Figure Legends:**

Figure S1. NS398 does not induce apoptosis in *Pkd1*^-/-^ cells. Representative images of Annexin V-FITC/PI apoptosis assay in *Pkd1*^-/-^ cells treated by vehicle and NS398.
